# Validation of the efficacy of the porous medium model in hemodynamic analysis of iliac vein compression syndrome

**DOI:** 10.3389/fbioe.2024.1481336

**Published:** 2025-01-06

**Authors:** Lingling Wei, Ke Hu, Jiaqiu Wang, Shuang Zhang, Xiaoxiao Yang, Yuanli Chen, Chenshu Li, Xinwu Lu, Kaichuang Ye, Peng Qiu, Yanqing Zhan

**Affiliations:** ^1^ Key Laboratory of Metabolism and Regulation for Major Diseases of Anhui Higher Education Institutes, Anhui Provincial International Science and Technology Cooperation Base for Major Metabolic Diseases and Nutritional Interventions, School of Food and Biological Engineering, Hefei University of Technology, Hefei, China; ^2^ School of Engineering, London South Bank University, London, United Kingdom; ^3^ Department of Vascular Surgery, The Affiliated Chuzhou Hospital of Anhui Medical University, Chuzhou, China; ^4^ Department of Vascular Surgery, Shanghai Ninth People’s Hospital Affiliated to Shanghai Jiao Tong University School of Medicine, Shanghai, China; ^5^ Department of General Surgery, The First Affiliated Hospital of Anhui Medical University, Hefei, China; ^6^ Department of General Surgery, Anhui Public Health Clinical Center, Hefei, China

**Keywords:** iliac vein compression syndrome, porous medium model, computational fluid dynamics, time to peak, clinical validation

## Abstract

Iliac Vein Compression Syndrome (IVCS) is a common risk factor for deep vein thrombosis in the lower extremities. The objective of this study was to investigate whether employing a porous medium model to simulate the compressed region of an iliac vein could improve the reliability and accuracy of Computational Fluid Dynamics (CFD) analysis outcomes of IVCS. Pre-operative Computed Tomography (CT) scan images of patients with IVCS were utilized to reconstruct models illustrating both the compression and collateral circulation of the iliac vein. A porous medium model was employed to simulate the compressed region of the iliac vein. The agreements of times to peak between discrete phase particles in CFD analysis and contrast agent particles in Digital Subtraction Angiography (DSA) were compared. Furthermore, comparisons were made between the CFD analysis results that incorporated the porous media and those that did not. The results revealed that in the CFD analysis incorporating the porous media model, more than 80% of discrete phase particles reached the inferior vena cava via collateral circulation. Additionally, the concentration variation curve of discrete phase particles demonstrated a high concordance rate of 92.4% compared to that obtained in DSA. In comparison to CFD analysis conducted without the porous medium model, the incorporation of the porous medium model resulted in a substantial decrease in blood flow velocity by 87.5% within the compressed region, a significant increase in pressure gradient of 141 Pa between the inferior vena cava and left iliac vein, and a wider distribution of wall shear stress exceeding 2.0 Pa in collateral vessels rather than in the compressed region. The study suggests that the introduction of a porous medium model improves the hemodynamic analysis of patients with IVCS, resulting in a closer alignment with clinical observations. This provides a novel theoretical framework for the assessment and treatment of patients with IVCS.

## 1 Introduction

Iliac Vein Compression Syndrome (IVCS) is a medical condition caused by the compression of the iliac vein or abnormalities in the vascular structure, resulting in impaired venous return from the lower limbs and pelvis (Poyyamoli et al., 2021). IVCS is a common risk factor for Deep Vein Thrombosis (DVT) in the lower limbs. DVT is characterized by abnormal blood clotting in the deep veins, severely impeding venous return and promoting collateral circulation between the iliac veins. Untreated DVT can lead to severe venous diseases in the lower limbs (Kalu et al., 2013; Virchows, 2024).

Clinical anatomical studies have identified a distinct fibrous structure, known as the venous ridge, in the compressed region of the iliac vein in IVCS patients (Zhou et al., 2022). This structure is primarily composed of collagen and elastin, and obstructs blood flow, contributing to the formation of DVT (Butros et al., 2013; Al-Otaibi et al., 2021). It is nearly invisible in medical imaging.

Collateral circulation refers to the development of alternative blood pathways through newly formed vessels when one iliac vein is blocked or narrowed. This allows blood to bypass the obstruction and return to the inferior vena cava (Chiu and Chien, 2011). The presence of collateral circulation helps maintain blood flow in the lower limbs and alleviate pressure in the narrowed iliac vein trunk. During the reconstruction of the iliac vein model in patients with IVCS, constructing a complete collateral circulation model is beneficial for conducting a comprehensive analysis of venous blood flow, thereby enhancing the accuracy of CFD analysis.

In recent years, there has been an increasing application of CFD in the analysis of venous blood flow. Assi et al. (2023) utilized CFD analysis to identify elevated shear rates in the compressed left iliac vein of IVCS patients. Jiang et al. (2021) investigated the impact of different collateral types and cross-sectional areas on the stenosis of the left iliac vein and distal deep vein thrombosis in IVCS patients, revealing that blood is more likely to stagnate at the site of iliac vein stenosis or its distal end, resulting in reduced flow velocity and volume. These studies suggest that CFD methods contribute to a better understanding of hemodynamic conditions during venous stenosis, by providing quantitative parameters such as velocity and pressure, as well as generating intuitive images and charts. CFD analysis of the iliac vein with established collateral circulation revealed an increase in flow and acceleration in velocity within the narrow vessel, attributed to the reduction in cross-sectional area at the compressed site (Li et al., 2023; Sandeep and Shine, 2021). However, clinical studies have shown that the vein crest and the establishment of collateral circulation result in limited and sluggish blood flow in the compressed area of the iliac vein. Therefore, it is imperative to introduce alternative models to enhance the accuracy of CFD analysis.

A Porous Medium (PM) is a material characterized by a skeletal framework and interconnected pores that facilitate fluid flow, resembling the structure and function of venous sinuses (Wang, 2000). The PM model serves as a mathematical representation of fluid flow and heat transfer within porous materials (Kaviany, 2012). Incorporating a PM model in the compressed region of the iliac vein in CFD analysis can better simulate the venous ridge structure, reducing blood flow velocity and volume in the compressed area (Bear and Bachmat, 1990). This aligns more closely with clinical scenarios and holds significant clinical relevance.

This study utilized Computed Tomography Venography (CTV) data from IVCS patients to reconstruct models. A PM model was employed to simulate the venous ridge structure and construct a complete collateral circulation pathway. Discrete phase particles were used to simulate the flow of contrast agents, and their times to peak (TTPs) in the inferior vena cava were monitored and compared with DSA to verify the feasibility and accuracy of the PM model in the hemodynamic analysis of IVCS. Additionally, comparing CFD results with and without the PM model explored the significance of the PM model in IVCS analysis, which provides a theoretical basis for clinical evaluation and treatment of IVCS.

## 2 Materials and methods

### 2.1 Data source

**FIGURE 1:**
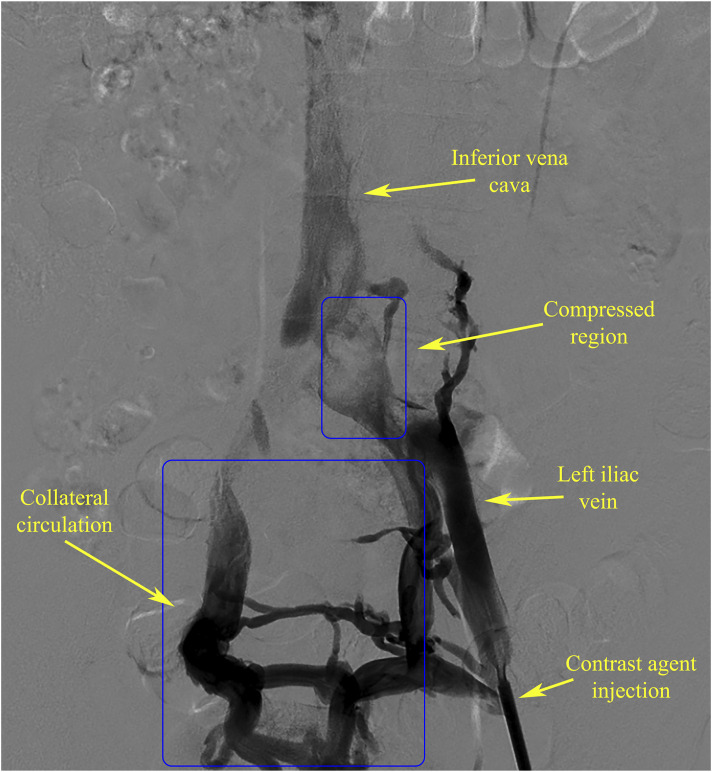
Clinical DSA images of the patient, indicating clear collateral circulation.

The DSA and CTV image data of an IVCS patient from the North District of the First Affiliated Hospital of Anhui Medical University were selected for analysis. The DSA data (Figure 1) showed severe compression of the left iliac vein, resulting in obstructed blood return and the presence of a well-developed collateral vascular pathway, which met the experimental requirements.

### 2.2 Model construction

**FIGURE 2:**
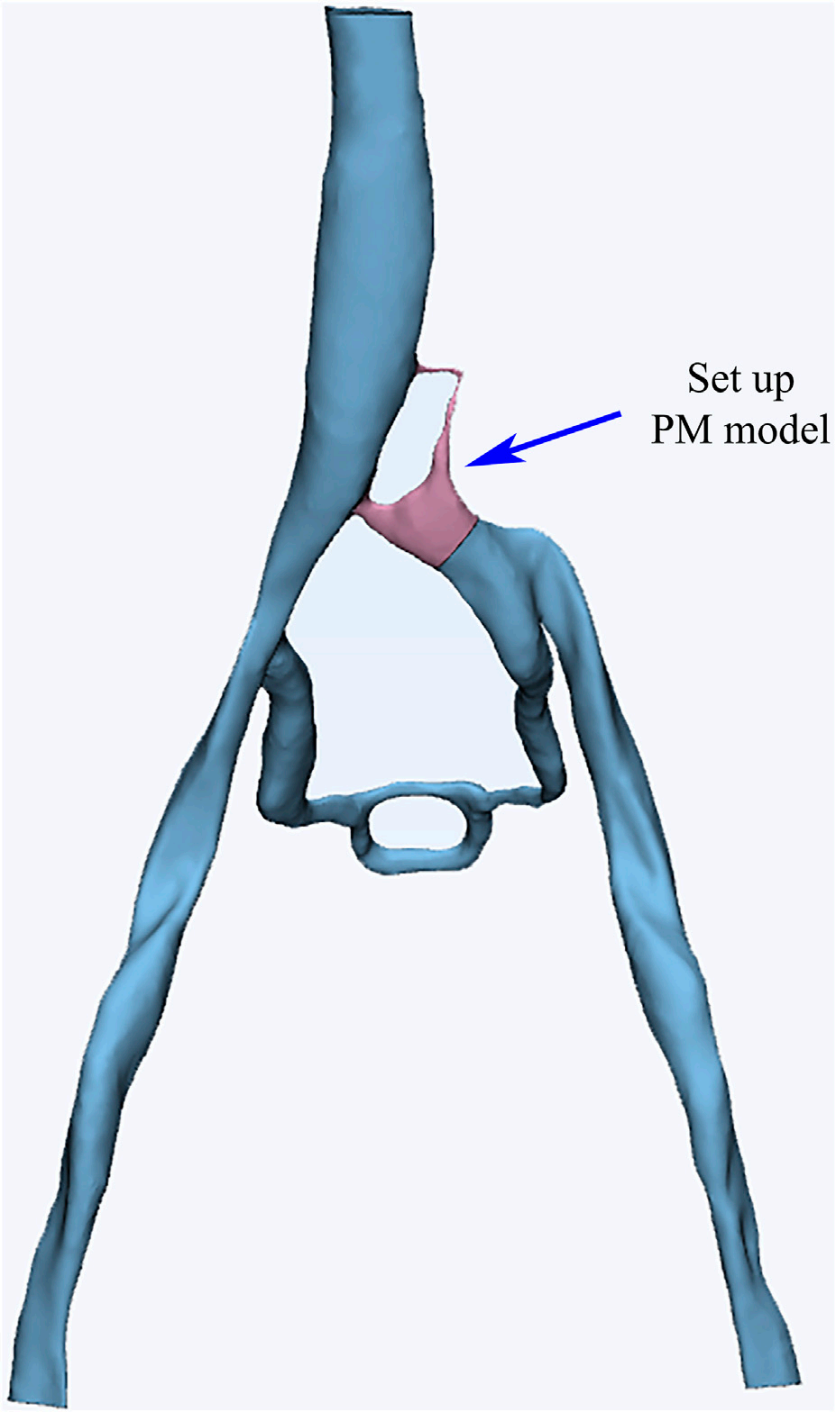
Reconstructed vascular model of the patient, with a set Porous Media model region.

The CTV data of the patient was utilized to reconstruct a 3D vascular model. The Mimics software was employed for color segmentation to extract the vessels, create centerlines, and fit the vascular spatial structure. To simplify the calculations, we excluded most of the smaller vessels that are not connected to the main veins during the vessel extraction process. These vessels typically branch off from the iliac vein and gradually diminish, disappearing into the tissue or capillaries, without affecting the primary blood flow of the iliac vein. Additionally, the 3-Matic software was used for segmentation, smoothing, and meshing to ultimately establish a 3D vascular model that included the left and right iliac veins, collateral vessels, and the inferior vena cava (Figure 2). Blood flow entered from both iliac veins, with most of the blood from the left iliac vein flowing through the collateral vessels to reach the right iliac vein before entering the inferior vena cava. Through DSA imaging and in collaboration with clinicians, the identified porous areas were strictly limited to regions characterized by compressed blood vessels.

### 2.3 CFD numerical simulation

The CFD analysis was conducted using ANSYS 22.0 software. The vascular walls were set as rigid and no-slip, with blood flow assumed to be laminar. Blood was modeled as a homogeneous, incompressible Newtonian fluid with a viscosity coefficient of 0.0029 kg/(m·s) and a density of 1,410 kg/m^3^.

Regarding the boundary condition settings for venous entrances, different approaches were employed for the left and right iliac veins. To simplify the analysis, a constant inlet velocity was assigned to the right iliac vein based on clinically measured averages, resulting in an established flow rate of 0.099 m/s. Conversely, the inlet velocity of the left iliac vein was defined as a variable and represented by a sine function that accurately fitted clinical ultrasound spectral data. This led to the following expression for its conditions: v = 0.108 + 0.0835 * sin (1.5t - 1.5) m/s. The outflow boundary condition was set to 0 Pa. For the CFD analysis, the coupled scheme was employed with a time step of 0.02 s, and transient calculations were performed for three cycles, totaling 14 s (Albadawi et al., 2022; Sikkandar et al., 2019; Taebi, 2022).

The PM model region was calculated using the Brinkman equation, which is suitable for incompressible Newtonian fluids with laminar flow in the pores. This calculation took into account both viscous and inertial resistance. The porosity and relative viscosity of the PM were assumed to be 1, and the viscous resistance coefficient was adjusted to identify the optimal combination of conditions.

**FIGURE 3:**
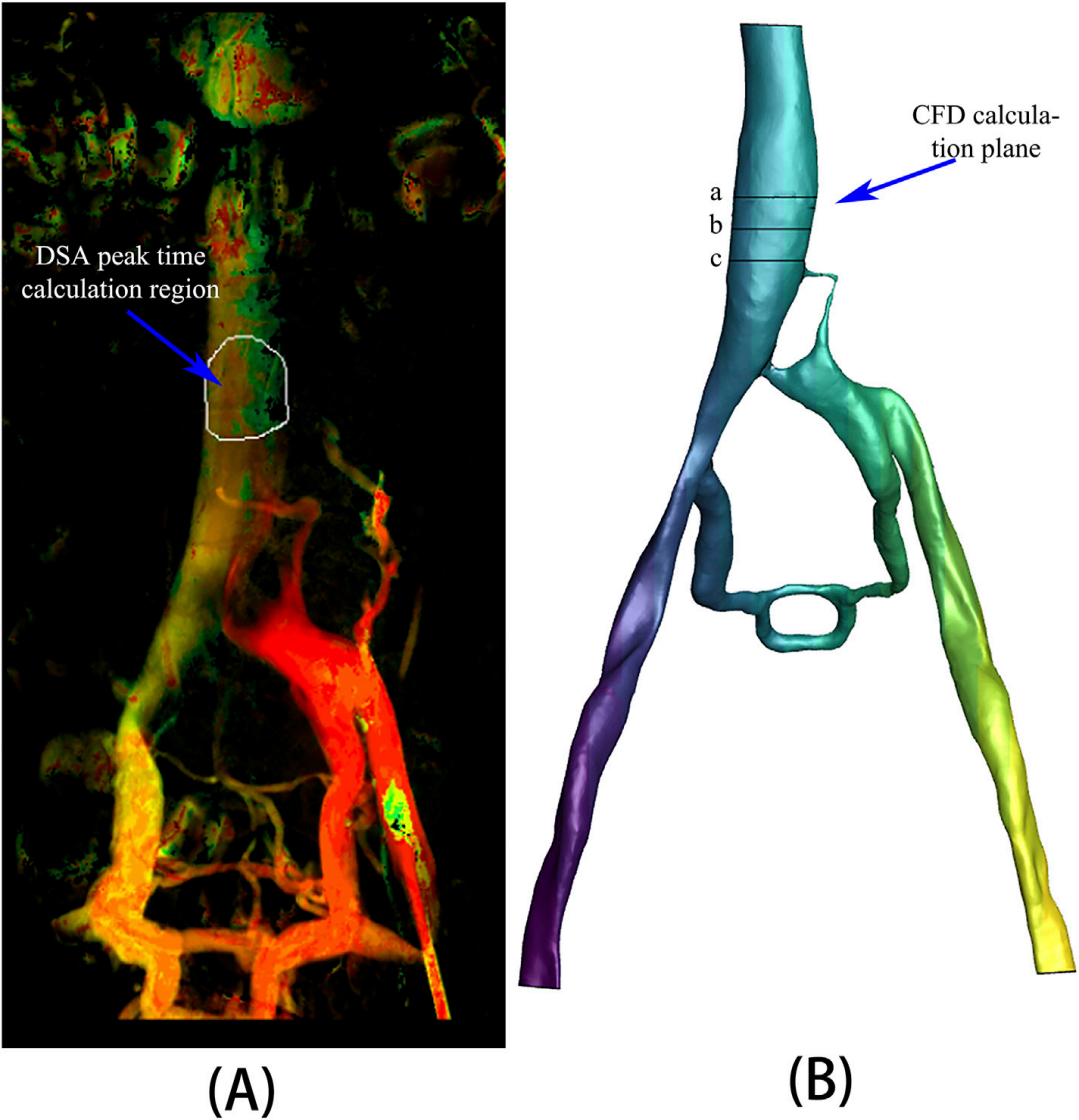
**(A)** Region selected for TTP calculation in DSA; **(B)** Calculation planes in CFD analysis.

A specific region of the inferior vena cava was chosen from the DSA image data to record the time to peak (TTP) of the contrast agent (Figure 3A). Correspondingly, three planes, spaced 10 mm apart, were selected at the appropriate location within the inferior vena cava model (Figure 3B) to calculate the mean values of surface integral velocities and particle counts. These values were utilized for grid independence verification and TTP calculations. TTP was defined as the duration from the start of contrast agent injection to its maximum concentration at a specified location, reflecting the vascular obstruction and collateral circulation condition in the patient.

The iliac vein model was meshed using tetrahedral elements. Mesh independence was verified by setting the triangular edge lengths to 0.23 mm, 0.30 mm, and 0.40 mm, resulting in 4 million, 2 million, and 1 million mesh elements, respectively. The surface integral of fluid velocity for these different mesh sizes were 0.02487 m/s, 0.02483 m/s, and 0.02495 m/s, respectively, with differences between each pair being less than 1%. Considering both mesh accuracy and computational cost, the 2 million-element mesh was selected for CFD analysis.

The Discrete Phase Model (DPM) is a CFD model utilized to simulate the movement of sparsely distributed particles (such as droplets, bubbles, solid particles, etc.) within a continuous phase (such as gas or liquid). It also accounts for the interaction between the discrete and the continuous phases. In this study, DPM was employed to simulate the flow of contrast agent particles in blood. An inert discrete phase model was utilized to simulate the movement and accumulation of the contrast agent within the blood vessels (Brandt and Coletti, 2021). The particle diameter was set to 0.001 mm, and particles were injected from a circular plane with a diameter of 1.5 mm at an injection rate of 0.02133 kg/s and an initial velocity of 0 m/s, following the blood flow. The calculation focused on simulating the flow of discrete phase particles from the left iliac vein inlet through the compressed region (i.e., the PM model region) and collateral vessels into the inferior vena cava. The quantity of discrete phase particles reaching the statistical plane of the inferior vena cava over time was then compared with the TTP of the contrast agent in DSA.

## 3 Results

### 3.1 Viscous resistance coefficient

Various viscous resistance coefficients for the PM model were selected based on a well-established range of parameters identified in the existing literature (Bear and Bachmat, 1990). The CFD analysis was performed using PM models with varying viscous resistance coefficients in order to assess the time of particles reaching the first peak, first valley, and second peak in comparison to results obtained in DSA (Table 1). The results indicated that three sets of data showed a TTP similarity of over 90% compared to DSA, and the viscous resistance coefficients were determined to be 1.0*10^6^, 5.0*10^7^, and 2.5*10^8^, respectively. Therefore, the PM model and viscous resistance coefficient can be used to evaluate the iliac vein compression in a patient.

**TABLE 1:** Comparison of TTPs among discrete phase particles in CFD analysis with different viscous resistance coefficients and contrast agent concentrations in DSA.

	Viscous resistance coefficient	First TTP (s)	First valley time (s)	Second TTP (s)	Average deviation	TTP similarity
0	DSA data	**2.75**	**4.30**	**5.70**		
1	1.0*10^6^	3.02	4.69	6.35	10.00%	90.00%
2	2.5*10^6^	2.88	3.92	10.2	31.03%	68.97%
3	5.0*10^6^	2.75	3.68	4.18	13.75%	86.25%
4	1.0*10^7^	3.13	3.95	7.68	18.82%	81.18%
5	2.5*10^7^	2.70	4.73	6.75	10.12%	89.88%
6	5.0*10^7^	2.59	4.02	6.36	7.97%	92.03%
7	1.0*10^8^	4.20	4.92	5.58	22.93%	77.07%
8	2.5*10^8^	3.47	4.45	5.79	7.57%	92.43%
9	5.0*10^8^	3.84	4.50	5.88	15.71%	84.29%

The bold values were obtained from DSA image data.

### 3.2 Time to peak (TTP)

**FIGURE 4:**
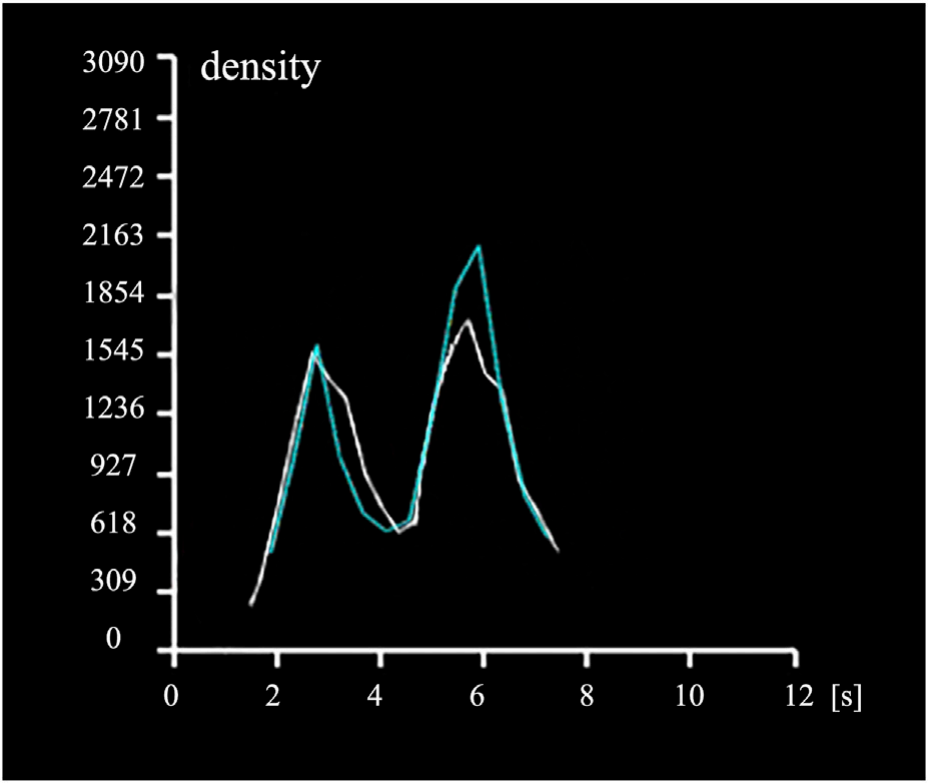
Time to Peak (TTP) curves. The white curve depicts the temporal variation of contrast agent concentration in the specified region of the inferior vena cava in DSA (Figure 3A), while the blue curve illustrates the temporal variation of discrete phase particle concentration in the specified plane of the inferior vena cava in CFD analysis (Figure 3B).

The number of discrete phase particles at a specified cross-sectional area of the inferior vena cava (Figure 3B) was counted and compared with the contrast agent particles observed in DSA. The concentration variation curve of discrete phase particles in the CFD analysis with a viscous resistance of 2.5*10^8^ best matched the concentration variation curve of contrast agent particles in DSA, as depicted in Figure 4. The TTP curve in the DSA was utilized to monitor concentration variations of contrast agent particles as they traveled from the left iliac vein, passed through the compressed region and collateral vessels, and ultimately converged in the inferior vena cava throughout the duration of the procedure. In the CFD analysis incorporating the PM model, the TTP curve was generated by employing discrete phase particles to simulate the behavior of the contrast agents. This approach enabled the simulation of concentration changes of discrete phase particles within a defined plane of the inferior vena cava.

From the graph, it can be observed that the discrete phase particles in the PM model and the contrast agent particles in DSA exhibited similar concentration variation trends. Both curves displayed double peaks, with peak times coinciding. In the PM model, the first TTP of discrete phase particle concentration was 3.465s, while the DSA showed the first TTP at 2.752s, resulting in a difference of 20.58%. The second TTP of concentration was achieved at 5.787s in PM model and at 5.702s in DSA, representing a difference of 1.47%. Additionally, the concentration reached a valley at 4.445s in PM model and at 4.304s in DSA, with a difference of 3.17%.

### 3.3 Velocity distribution of phase discrete particles

**FIGURE 5:**
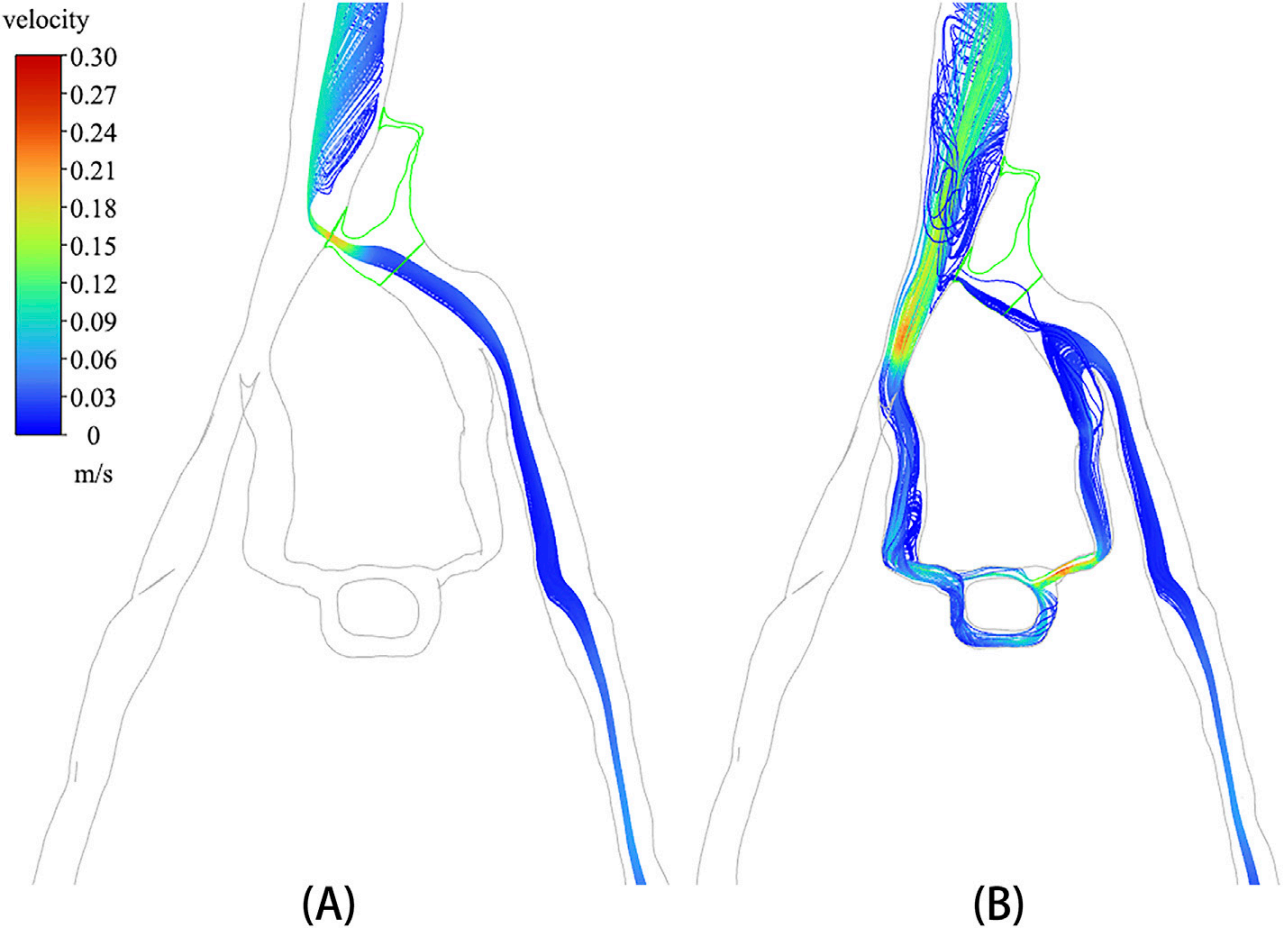
Velocity distribution of discrete phase particles: **(A)** NPM model; **(B)** PM model.

A discrete phase particle model was employed to simulate the injection of contrast agents from the left iliac vein. Figure 5 displays the particle distributions in both the non-porous medium (NPM) model and the PM model. The distribution of discrete phase particles in the PM model showed a notable similarity to the contrast agents in DSA, indicating that the PM model provides a more precise representation of venous flow characteristics in IVCS patients. A significant difference was observed in the velocity and distribution of particles within the compressed region of the iliac vein between the two models. In the NPM model, all discrete phase particles flowed into the inferior vena cava through the compressed region, displaying a notably increased velocity within this area. Conversely, in the PM model, over 80% of the discrete phase particles entered the inferior vena cava through collateral vessels, with only a small number of particles passing through the compressed region at a slower velocity.

**FIGURE 6:**
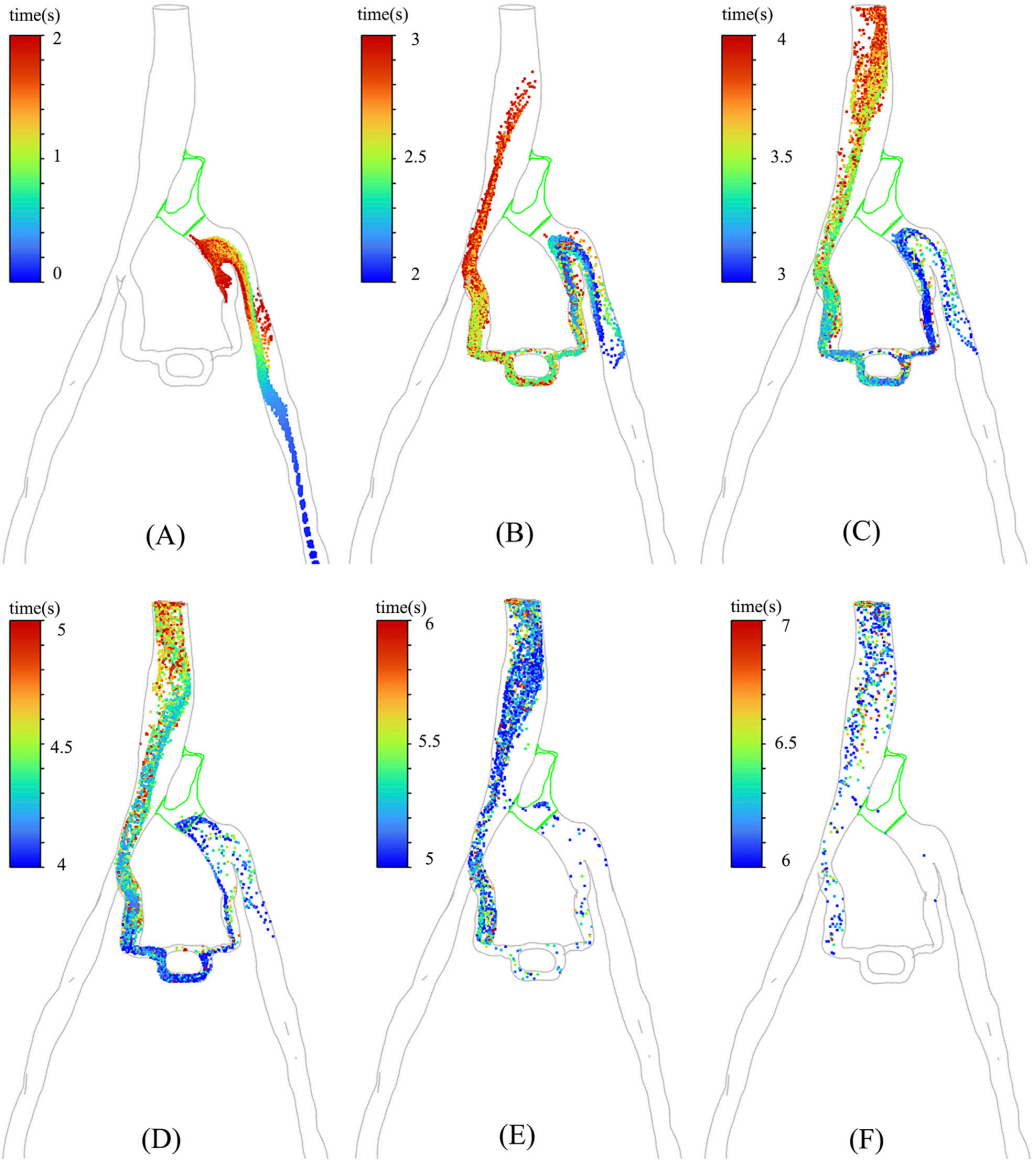
The distribution of particles at different moments in PM model: **(A)** 0–2 s; **(B)** 2–3 s; **(C)** 3–4 s; **(D)** 4– ; **(E)** 5–6 s; **(F)** 6–7 s.

In particular, the temporal distribution of particles in CFD analysis with incorporated PM model is illustrated in Figure 6. It is shown that from 0 s to 7 s, the temporal distribution of particles is well aligned with the TTP curve depicted in Figure 4. Figure 6 revealed that a substantial number of discrete phase particles traveled through the collateral vessels into the inferior vena cava over time, while only a minimal quantity of particles passed through the compressed region. This pattern closely resembled the movement of the contrast agents observed in clinical practice, supporting the notion that both the porous media model and the discrete phase model could effectively simulate the dynamics of the contrast agent within the compromised vascular environment. These findings highlight the potential utility of these modeling approaches in enhancing the understanding of hemodynamics in patients with IVCS.

### 3.4 Pressure gradient

**FIGURE 7:**
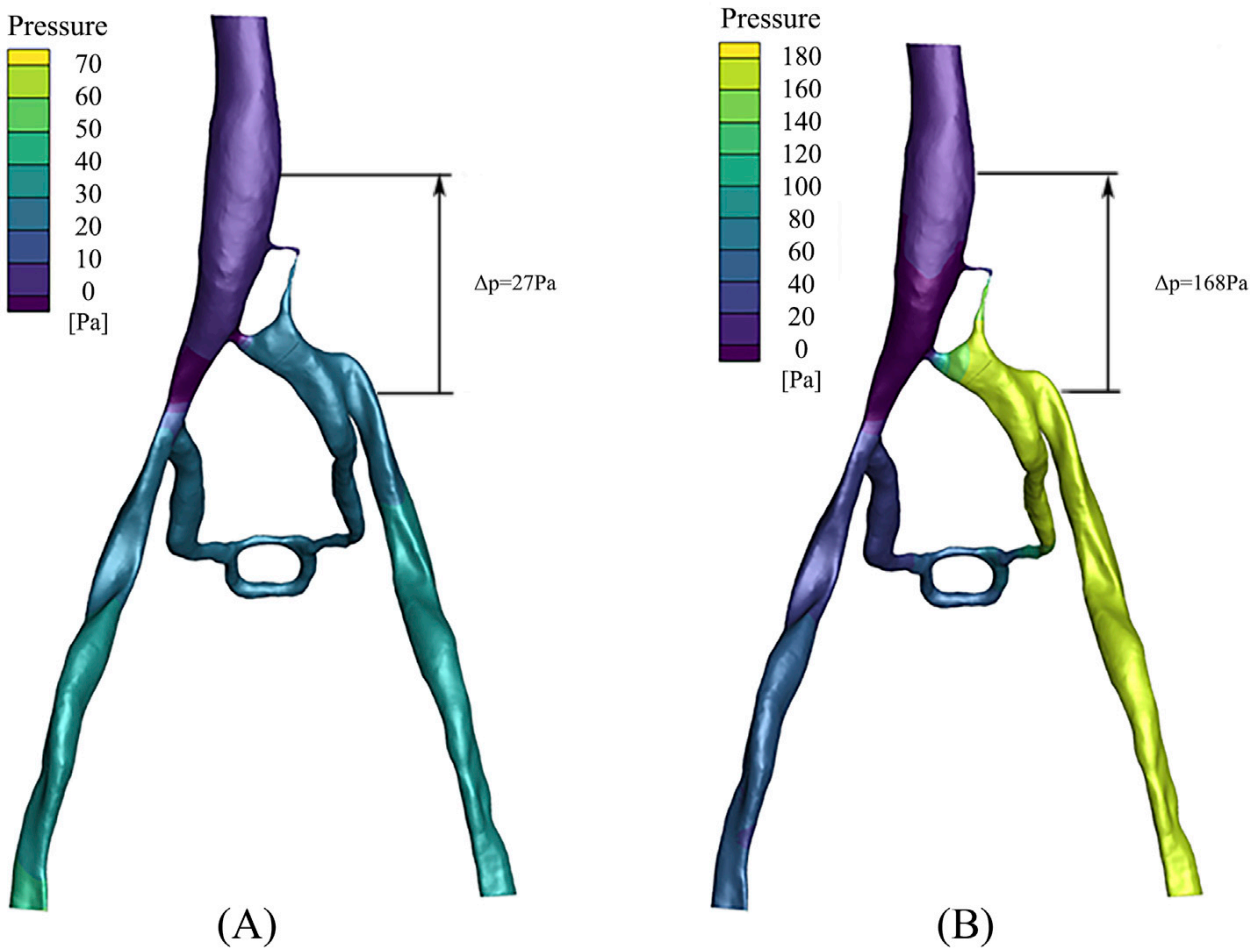
Pressure distribution: **(A)** NPM model; **(B)** PM model.

The pressure distribution in the PM and NPM models is depicted in Figure 7. As illustrated, the pressure in the left iliac vein of the NPM model ranged from 20 to 30 Pa, whereas in the PM model, it varied from 160 to 180 Pa, indicating a higher pressure in the left iliac vein. Within the porous medium region (i.e., the stenosis area of the iliac vein), the maximum pressure in the NPM model was 28 Pa, whereas it was 167 Pa in the PM model.

In clinical practice, the pressure gradient is commonly utilized to assess the severity of a patient, which is defined as the pressure difference between the inferior vena cava and the left iliac vein. The data revealed that in the NPM model, the pressure gradient across the compressed region was 27 Pa, whereas it increased to 168 Pa in the PM model. The pressure gradient in the PM model exceeded that of NPM by 141 Pa, demonstrating a closer approximation to clinical reality.

### 3.5 Wall shear stress

**FIGURE 8:**
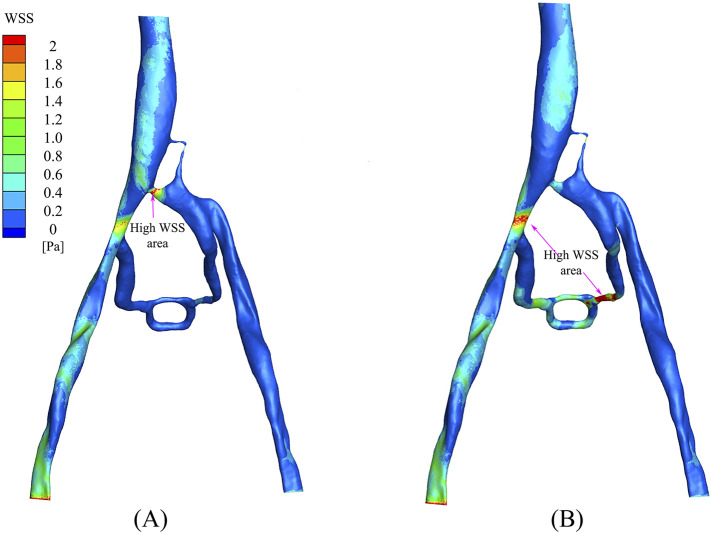
WSS distribution: **(A)** NPM model; **(B)** PM model.

The distribution of wall shear stress (WSS) in the PM and NPM models is shown in Figure 8. WSS serves as a crucial parameter that indicates the health status of the vascular wall and blood cells (Candreva et al., 2024). In arterial studies, WSS values above 2.0 Pa are generally deemed to be detrimental to the vessel wall, whereas WSS values below 0.5 Pa are associated with thrombosis risk (Eshtehardi et al., 2012; Kumar et al., 2018; Samady et al., 2011). In the NPM model, there were limited regions with WSS exceeding 2.0 Pa, predominantly concentrated at the junction of the compressed region and the inferior vena cava. In contrast, the PM model displayed a wider distribution of areas with WSS surpassing 2.0 Pa, primarily situated in the collateral vessels and their junction with the right iliac vein. Furthermore, in the NPM model, regions characterized by low WSS (below 0.5 Pa) were distributed throughout the left iliac vein and collateral vessel regions, while in the PM model, low WSS regions were confined to the vicinity of the left iliac vein.

## 4 Discussion

In the hemodynamic study of the iliac vein, traditional CFD analyses often overlook the structure of venous ridges in patients with iliac vein compression syndrome (IVCS), resulting in discrepancies between computational results and clinical observations. This study employed the PM model to simulate the function and characteristics of venous ridges and validated the feasibility of the PM model in IVCS hemodynamic analysis through TTP.

The TTP of discrete phase particles in CFD analysis incorporating the PM model was found to be highly consistent with that of contrast agents in DSA. This can be attributed to several reasons: firstly, the utilization of the PM model resulted in a reduction of velocity for discrete phase particles within the compressed region, thereby extending the time for particles to reach the inferior vena cava; secondly, the application of the PM model led to an increase of blood flow in collateral circulation, thereby extending the physical distance for particles to travel to the inferior vena cava. The strong resemblance observed between the results of CFD analysis and DSA indicates that the PM model exhibits similar flow characteristics to the compressed iliac vein. Therefore, the PM model can serve as a valuable tool for simulating the venous ridge structure in patients with IVCS. By adjusting the viscosity coefficient of the Porous Media, the PM model can offer a more precise representation of the compressed iliac vein, thereby providing a new theoretical framework for the diagnosis and treatment of IVCS cases.

In comparison to the NPM model, the PM model demonstrated a decrease in the flow and velocity of discrete phase particles within the compressed region, with most particles entering the inferior vena cava through collateral vessels. The reduction in particle velocity within the compressed region in the PM model was primarily attributed to two factors. Firstly, the PM model simulated the compressed region as a porous medium, which increased resistance to particle flow and consequently reduced their velocity. Secondly, the PM model enhanced the blood flow in collateral circulation, resulting in fewer particles entering the inferior vena cava through the compressed region, thereby reducing the velocity of particles within the compressed area. This phenomenon occurs specifically in patients with fully developed collateral circulation. Chen et al. (2023) found that patients with IVCS and without collateral vessels exhibited an increase in blood flow velocity within the compressed region as the severity of iliac vein stenosis progressed. However, Assi et al. (2023) demonstrated that the presence of collateral vessels significantly reduced blood flow through the left iliac vein to the inferior vena cava by over 50%. These studies suggest that the presence of collateral circulation affects the hemodynamic pattern of the iliac vein in patients with IVCS and can effectively redirect blood flow away from the compressed area towards the inferior vena cava. Compared with the NPM model, the PM model can better simulate this hemodynamic feature. In simulating iliac vein compression with collateral circulation, the PM model can restore real blood flow characteristics by allowing more blood flow into inferior vena cava through the collateral vessels instead of the compressed region, which is more closely aligns with clinical reality.

When comparing the pressure gradient of the left iliac vein and inferior vena cava between PM and NPM models, it was found that the pressure gradient in the PM model was significantly higher than in the NPM model. This finding is consistent with previous research by Li et al. (2023), which demonstrated a notable increase in pressure gradient between these two locations as IVCS progressed. A higher pressure gradient can serve as an indicator of the severity of IVCS, with a pressure gradient exceeding 266 Pa commonly seen as a clinical characteristic of this condition (Liu et al., 2014; O'Sullivan et al., 2000). Although the pressure gradient in PM model did not reach 266 Pa in this study, it is clear that there is a tendency for the PM model to cause a greater pressure gradient difference. The failure to achieve 266 Pa may be attributed to the fact that patients with IVCS in this study were affected by a combination of other factors and pressure gradients. When compared to the NPM model, it is evident that an increased pressure gradient in the PM model aligns more closely with clinical observations.

The comparison of wall shear stress (WSS) between the PM and NPM models revealed that the PM model demonstrated a larger region of high WSS (exceeding 5 Pa). Elevated high WSS regions typically indicate an increased risk of vascular injury in arterial studies (Fallahi et al., 2021; Sandeep and Shine, 2021). A study by Li et al. (2023) demonstrated that an increase in iliac vein stenosis led to a rise in high WSS regions The higher proportion of high WSS regions in the PM model is more consistent with clinical observations. Conversely, when analyzing low WSS regions, the NPM model showed a larger region with low WSS levels, primarily concentrated in the collateral vessel area. This discrepancy may be attributed to the limited blood flow distribution in the collateral vessels within the NPM model.

This study demonstrates that the incorporation of a porous medium model significantly enhances the hemodynamic analysis of patients with IVCS, leading to a hemodynamic environment that more closely aligns with clinical observations. This improvement is attributed to the effective simulation capabilities of the PM model regarding the obstructive effects posed by venous ridge structures in IVCS. Consequently, when compressed tissues generate ridge-like formations within the constricted region of a vessel, the PM model could accurately replicate the resultant flow disturbances. Therefore, this approach holds considerable promises for conditions such as inferior vena cava compression syndrome or jugular vein compression syndrome.

In this research, a PM model was established in the compressed region of iliac vein, ensuring that parameters such as blood flow velocity, pressure gradient, and wall shear stress in CFD analysis accurately reflected clinical realities. Such approach facilitates a more precise evaluation of both the degree and specific locations of iliac vein compression. Such insights not only provide robust support for disease diagnosis and treatment, but also play a crucial role in preventing complications, evaluating treatment efficacy, and assessing prognosis. Furthermore, conducting precise CFD analysis of IVCS with the incorporated PM model, could elucidate the relationship between hemodynamic characteristics of IVCS and its onset and progression. This investigation may reveal the pathophysiological mechanisms underlying this condition, thereby providing a theoretical foundation and experimental support for the development of novel therapeutic strategies.

This study has several limitations. Firstly, the sample size was relatively small, which restricts the generalizability of the findings, necessitating further expansion to verify the feasibility of the porous medium model. A larger sample size would not only enhance the statistical power of the study but also facilitate a more nuanced understanding of the performance across diverse patient demographics. This is particularly important given the variability in anatomical and physiological characteristics among individuals with IVCS. Secondly, this study only considered a single porous medium model without investigating the performance differences between various model configurations. Future research could benefit from the selection or design of multiple porous medium models tailored to different patient characteristics, thereby improving both applicability and accuracy of the PM model. Lastly, to further validate the accuracy of the simulation results, it is crucial to conduct *in vitro* or *in vivo* experiments. Such investigations will help confirm validity of the model and provide new theoretical support for the treatment of IVCS patients, ultimately enhancing clinical outcomes.

## 5 Conclusion

In this study, the PM model was utilized to simulate the compressed region of the iliac vein in patients with IVCS, and the feasibility of this model was validated through the hemodynamic analysis of IVCS. The results demonstrated that CFD analysis with the PM model could replicate the hemodynamic environment of IVCS patients. Incorporating the PM model in CFD analysis reduced the flow and velocity in the compressed iliac vein, altered the distribution of blood flow, and directed most of the blood towards the right iliac vein through collateral circulation before returning to the inferior vena cava. Additionally, the pressure gradient value between inferior vena cava and left iliac vein closely approximated to clinical pressure gradient index. Therefore, CFD analysis using PM model has potential applications for evaluating IVCS patient conditions and offering valuable insights for the diagnosis and treatment of IVCS from a fluid mechanics perspective.

## Data Availability

The original contributions presented in the study are included in the article/supplementary material, further inquiries can be directed to the corresponding authors.
